# Development of a Tumor-Selective Approach to Treat Metastatic Cancer

**DOI:** 10.1371/journal.pone.0000023

**Published:** 2006-12-20

**Authors:** Karen S. Aboody, Rebecca A. Bush, Elizabeth Garcia, Marianne Z. Metz, Joseph Najbauer, Kristine A. Justus, Doris A. Phelps, Joanna S. Remack, Karina Jin Yoon, Shanna Gillespie, Seung U. Kim, Carlotta A. Glackin, Philip M. Potter, Mary K. Danks

**Affiliations:** 1 Divisions of Hematology/Hematopoietic Cell Transplantation and Neurosciences, and Department of Professional Education, City of Hope National Medical Center Duarte, California, United States of America; 2 Department of Molecular Pharmacology, St. Jude Children's Research Hospital Memphis, Tennessee, United States of America; 3 Department of Medicine, University of British Columbia Hospital Vancouver, British Columbia, Canada; 4 Ajou University School of Medicine Suwon, South Korea; Utrecht University, Netherlands

## Abstract

**Background:**

Patients diagnosed with metastatic cancer have almost uniformly poor prognoses. The treatments available for patients with disseminated disease are usually not curative and have side effects that limit the therapy that can be given. A treatment that is selectively toxic to tumors would maximize the beneficial effects of therapy and minimize side effects, potentially enabling effective treatment to be administered.

**Methods and Findings:**

We postulated that the tumor-tropic property of stem cells or progenitor cells could be exploited to selectively deliver a therapeutic gene to metastatic solid tumors, and that expression of an appropriate transgene at tumor loci might mediate cures of metastatic disease. To test this hypothesis, we injected HB1.F3.C1 cells transduced to express an enzyme that efficiently activates the anti-cancer prodrug CPT-11 intravenously into mice bearing disseminated neuroblastoma tumors. The HB1.F3.C1 cells migrated selectively to tumor sites regardless of the size or anatomical location of the tumors. Mice were then treated systemically with CPT-11, and the efficacy of treatment was monitored. Mice treated with the combination of HB1.F3.C1 cells expressing the CPT-11-activating enzyme and this prodrug produced tumor-free survival of 100% of the mice for >6 months (*P*<0.001 compared to control groups).

**Conclusions:**

The novel and significant finding of this study is that it may be possible to exploit the tumor-tropic property of stem or progenitor cells to mediate effective, tumor-selective therapy for metastatic tumors, for which no tolerated curative treatments are currently available.

## Introduction

Neuroblastoma is the most common extracranial solid tumor in children. Typically, patients diagnosed with high-risk disease demonstrate a good initial response to therapy, but as many as 80% of these patients relapse with metastatic disease that is refractory to therapy. Like other solid tumors, when neuroblastomas metastasize, they are very difficult to treat, and a majority of children with metastatic neuroblastoma die of their disease [1]–[3]. Currently available treatments have anti-tumor efficacy but they also produce undesirable side effects to normal tissue, limiting the treatment that can be administered. Novel and effective approaches for the treatment of neuroblastoma are needed.

The approach described in this study is based on exploiting the tumor-tropic property of HB1.F3.C1 cells [4]–[16] to deliver an effective therapeutic agent selectively to tumors. The specific goal of the study was to show that intravenous administration of HB1.F3.C1 cells expressing the CPT-11 (irinotecan)-activating enzyme rabbit carboxylesterase (rCE) would significantly increase the antitumor effect of tolerated doses of CPT-11 in mice bearing disseminated neuroblastoma tumors. The approach described might also be adapted to developing treatment for patients with other types of metastatic solid tumors.

Neural stem cells (NSCs) or progenitor cells (NPCs) and mesenchymal stem cells (MSCs) have recently been investigated as delivery vehicles for treating subcutaneous xenografts, as well as for treating tumors in the central nervous system or lungs of preclinical models. This is the first study attempting to exploit the remarkable tumor-tropism of these cells to develop treatments for metastatic, disseminated solid tumors. Since no effective treatments are available for most metastatic tumors, demonstrating that stem or progenitor cells of fetal or adult origin can be used to improve the prognosis of patients with fatal metastatic disease would be highly significant.

The cell line used in this study (HB1.F3.C1) was derived from fetal telencephalon cells. Primary cells were immortalized by retroviral insertion of the v-*myc* gene which was required to sustain their replication potential [17]. Following immortalization, HB1.F3.C1 cells replicate *in vitro* to form identical daughter cells or can be induced to differentiate into other cells of neuronal lineage, thereby exhibiting characteristics of both stem cells and progenitor cells. Since HB1.F3.C1 cells administered systemically migrate to and localize *in vivo* at sites of pathology including tumors, we proposed to exploit this property to selectively deliver rCE to activate the anticancer prodrug CPT-11 [18]–[20]. We hypothesized that increased conversion of this prodrug to its active metabolite (SN-38) at tumors sites would increase selective antitumor activity. We administered intravenously adenovirus-transduced HB1.F3.C1 cells expressing a secreted form of rCE to mice bearing disseminated neuroblastoma. Mice were then treated systemically with CPT-11 and long-term survival of animals was monitored. The results obtained suggest that novel enzyme/prodrug approaches to therapy using stem or progenitor cells as delivery vehicles may have utility in the treatment of metastatic cancer.

## Methods

### Tumor Cell Lines

The three human neuroblastoma cell lines used in this study were NB-1691, NB-1643, and SK-N-AS [21], [22]. These cell lines were obtained from the Pediatric Oncology Group and the American Type Culture Collection and reflect different neuroblastoma phenotypes and genotypes: N-*myc* amplified, N-*myc* non-amplified/overexpressed, N-*myc* non-amplified/low level expression, *MDM2* amplified, and different ratios of nuclear/cytoplasmic p53. Each type of experiment detailed below was done with all three cell lines; results from representative experiments are shown. Three neuroblastoma cell lines were used to show that results observed were not limited to a single cell line; similar results were observed with all three cell lines. Tumor cell lines were grown in DMEM containing 10% FCS at 37°C in a humidified atmosphere containing 10% CO_2_.

### HB1.F3.C1 Cell Line

The HB1.F3.C1 cell line is a multipotent, cloned cell line that was generated by immortalizing cells obtained from the telencephalon of a human fetus of 15 weeks' gestation, using a retrovirus encoding the v-*myc* gene [17], [23]. The primary cells were obtained in accordance with the Guidelines of the Anatomical Pathology Department of Vancouver General Hospital, with permission to use fetal tissue granted by the Clinical Research Screening Committee Involving Human Subjects of the University of British Columbia. HB1.F3.C1 is an established, well-characterized, stable cell line [23]–[27]. HB1.F3.C1 cells self-renew *in vitro*, are non-tumorigenic, and are multipotent in that they can be induced to differentiate into neurons, oligodendrocytes, and astrocytes [17]. The use of this cell line circumvented the significant problem of limited availability of large quantities of primary cells and maximized reproducibility among experiments. The cell line was grown in DMEM with 10% FCS at 37°C in a humidified atmosphere of 10% CO_2_.

### Transduction of HB1.F3.C1 Cells to Express rCE

Replication-deficient adenovirus expressing a secreted form of rCE under the control of the cytomegalovirus (CMV) promoter (AdCMVrCE) was constructed using standard methods, as described previously [18], [20], [28]. HB1.F3.C1 cells were co-cultured with AdCMVrCE for 24 hours prior to intravenous injection. The enzyme activity assay used to quantitate the level of expression of rCE by HB1.F3.C1 cells transduced with adenovirus has also been reported previously [20].

### HB1.F3.C1 Cell Migration, Injection and Localization

To examine whether HB1.F3.C1 cells, injected intravenously, migrate to disseminated tumor foci in mice, neuroblastoma tumor cells were injected intravenously into the tail veins of mice and allowed to seed and develop tumors for two months. HB1.F3.C1 cells (2×10^6^) were then injected *via* the same route and organs were harvested 3–4 days later to evaluate migration to macroscopic and microscopic tumors.

For therapeutic experiments, HB1.F3.C1 cells were administered 2 weeks following injection of neuroblastoma cells. At this time point, tumors are microscopic and undetectable by eye or by *in vivo* imaging. Consistent with initiating treatment at this time point, we propose that the most likely eventual clinical utility of the described approach will be to eradicate minimum residual disease following conventional therapy. This experimental protocol most directly reflects that potential application.

In both migration and therapeutic studies, 2×10^6^ HB1.F3.C1 cells transduced with adenovirus at a multiplicity of infection (MOI) of 20 were pre-labeled with CM-DiI (chloromethylbenzamido-1,1′-dioctadecyl-3,3,3′,3′-tetramethylindocarbocyanine perchlorate, Molecular Probes, Eugene, OR) according to manufacturer's instructions. Cells were then rinsed 3 times with PBS and injected into the tail vein of tumor-bearing mice. CM-DiI was chosen because it is non-diffusible by virtue of its covalent binding to cellular thiols, and has been shown to be suitable for labeling and *in vivo* tracking of cells for at least 10 weeks [29], [30]. In experiments included in this paper, mice injected with CM-DiI-labeled cells were harvested 3–4 days following labeling and injection.

### Es1^e^ Esterase-deficient Severe Combined Immunodeficient (SCID) Mice

The plasma of SCID mice contains high levels of a rodent carboxylesterase that activates CPT-11 [31], [32]. Therefore, we used esterase-deficient SCID mice for all *in vivo* therapeutic studies [33]. These animals have levels of plasma esterase comparable to those in human plasma. Mice were housed in an AALAAC-accredited facility and were given food and water ad libitum.

### RT-PCR/PCR

Bone marrow was harvested from the tibia of normal and tumor-bearing mice, and RNA was extracted for RT-PCR detection of tyrosine hydroxylase (TH), a neuroblastoma cell marker. DNA was extracted from a separate aliquot of bone marrow for PCR of the v-*myc* gene to identify HB1.F3.C1 cells. Primer sequences and RT-PCR conditions for TH have been published previously [21]. Standard methods were used for v-*myc* amplification using a forward primer of 5′-CCTTTGTTGATTTCGCCAAT-3′ and a reverse primer of 5′-AGTTCTCCTCCTCCTCCTCG-3′, with an annealing step of 62.5°C for 1 minute for 30 cycles. The positive control for TH was RNA extracted from NB-1691 cells, and for the v-*myc* gene was DNA from HB1.F3.C1 cells. Negative controls contained no RT or no DNA, for TH and v-*myc* reactions, respectively.

### Histochemistry, Immunohistochemistry and Immunofluorescence Imaging

Organs were harvested, fixed in 4% paraformaldehyde/PBS, pH 7.4 and cryoprotected in 30% sucrose. Fixed tissues were then embedded in OCT, serially cryosectioned (10 µm), thaw-mounted onto glass slides and stored dry at −20°C. For routine histological screening, tissue sections were stained with hematoxylin and eosin by standard methods.

Presence of HB1.F3.C1 cells at neuroblastoma tumor loci or in normal tissue was examined using fluorescence microscopy. In sections prepared as detailed above, nuclei of all cells were stained with DAPI (4′,6-diamidino-2-phenylindole; Sigma Biochemical, St. Louis, MO; blue fluorescence), and detected using epifluorescence excitation/emission filters of 340–380/420 nm (LP) (UV-2A, Nikon). HB1.F3.C1 cells, labeled with CM-DiI (red fluorescence), were detected using excitation/emission filters of 540–580 nm and 600–660 nm (Y-2E/C), using a Nikon Eclipse TE2000-U microscope (Nikon Instruments, Melville, NY) equipped with a SPOT RT Slider digital camera (Diagnostic Instruments, Sterling Heights, MI). To detect human neuroblastoma tumors in mouse tissues, we performed immunohistochemical staining using a mouse monoclonal antibody that recognizes human mitochondrial protein (Chemicon, Temecula, CA). Sections were post-fixed in 4% paraformaldehyde for 10 min, rinsed, permeabilized with 0.3% Triton X-100/PBS, 30 min and incubated in blocking solution (5% BSA+3% normal horse serum+0.1% Triton X-100, 1 h). Sections were then incubated sequentially with primary antibody (1∶100 dilution) for 16 h at 4°C, biotinylated anti-mouse IgG secondary antibody (Vector Laboratories, Burlingame, CA) for 2 hours, and avidin-FITC (Vector Laboratories) for 1 hour. Tissue sections were counterstained with DAPI and mounted with fluorescent mounting medium (DAKO). The FITC fluorescence was detected by epifluorescence filter (465–495 nm excitation, 515–555 nm emission; B-2E/C).

For routine histological evaluation of presence of tumors in various organs, tissue sections were stained with hematoxylin and eosin. Adjacent sections were processed for immunoperoxidase-3,3′-diaminobenzidine (DAB) staining similarly as tissues processed for fluorescence staining, except that after permeabilization, endogenous peroxidases were quenched with 0.3% hydrogen peroxide/PBS for 30 min. Antibody reactivity to human mitochondria was subsequently detected using a Vectastain ABC *Elite* kit (Vector Laboratories) and a Peroxidase Substrate Kit (Vector Laboratories) according to the manufacturer's directions.

Low- and high-magnification images were obtained using a Nikon Eclipse TE2000-U microscope (Nikon Instruments, Melville, NY) equipped with brightfield and fluorescence illumination. Images were recorded and stored using SPOT Advanced and Adobe Photoshop software.

### Modified Boyden Chamber Cell Migration Assay

Modified Boyden chamber assays were used to assess the *in vitro* tropism of HB1.F3.C1 cells to tumor cell-conditioned media or to control media, using standard methods. Briefly, HB1.F3.C1 cells transduced with a MOI of 0, 5, 10 or 20 AdCMVrCE were trypsinized, washed, and resuspended in DMEM containing 5% BSA. Cells (3×10^4^ cells/100 µl) were placed into upper chambers and neuroblastoma cell-conditioned medium in lower chambers. Neuroblastoma cell-conditioned medium in both the upper and lower chambers comprised the chemokinesis control without a chemoattractant gradient. Cells were allowed to migrate for 4 hours in a cell culture incubator at 37°C and 5% CO_2_. The number of migrated HB1.F3.C1 cells in the lower chamber of each well was quantitated using CyQuant GR fluorescent dye (Chemicon) and a fluorescence microplate reader (Molecular Devices, Sunnyvale, CA). Assays were performed in triplicate.

### Animal Studies

Disseminated neuroblastoma was produced by injecting 5×10^5^ NB-1691, NB-1643, or SK-N-AS cells into the tail veins of mice. Typically these mice require euthanasia ∼75 days following injection of neuroblastoma cells. Gross tumors in multiple organs are visible at necropsy. This metastatic neuroblastoma model has been well characterized and is highly reproducible with regard to the course of disease and organ involvement [34]. In addition, the model recapitulates the clinical pattern of metastatic neuroblastoma in that tumors develop in multiple loci, including the adrenal gland, bone marrow and liver.

Each treatment group included 10 mice, monitored daily for the presence of disease or ill health. The weekly schedule of administration of HB1.F3.C1 cells and CPT-11 (7.5 mg/kg) is illustrated in Figure 8. This regimen was administered for 2 consecutive weeks, followed by a 2-week rest period and then another 2-week course of therapy. All animal protocols were approved by the City of Hope or the St. Jude Children's Research Hospital IACUC. When mice appeared to be in discomfort or distress as judged by independent animal care personnel with no knowledge of the protocol design, animals were euthanized. All euthanized mice were verified as tumor-bearing by necropsy. The endpoint for the therapeutic study was long-term survival.

**Figure 1:**
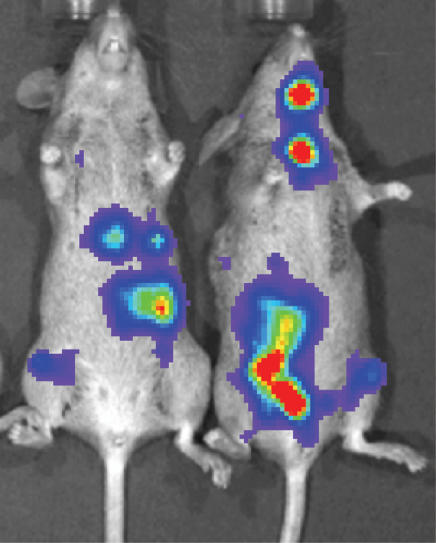
Es1^e^ SCID mice injected intravenously with neuroblastoma cells develop multiple disseminated tumors. SK-N-AS cells (5×10^5^) transduced to express luciferase were injected into tail veins. One month following injection of tumor cells, mice were injected intraperitoneally with luciferin and imaged using a Xenogen IVIS imaging system, according the directions of the manufacturer. Multiple tumors were present in 100% of mice. Two representative mice are shown.

**Figure 2:**
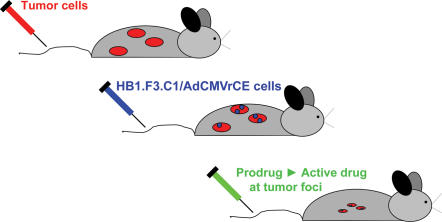
Schematic diagram of the protocol for NDEPT. Human neuroblastoma tumor cells are injected intravenously to produce disseminated tumors. At an appropriate time after injection of neuroblastoma cells, neural stem cells or neural progenitor cells transduced with adenovirus to express a prodrug-activating enzyme (in this study, a secreted form of rabbit carboxylesterase [rCE]) are injected intravenously. Following migration of stem cells or progenitor cells to tumor foci and a delay of 3–4 days to allow relatively high level expression of the prodrug-activating enzyme into the extracellular milieu at the tumor sites, mice are treated with the prodrug (in this study, CPT-11). The prodrug is activated selectively at tumor foci, to increase the therapeutic index of the prodrug.

**Figure 3:**
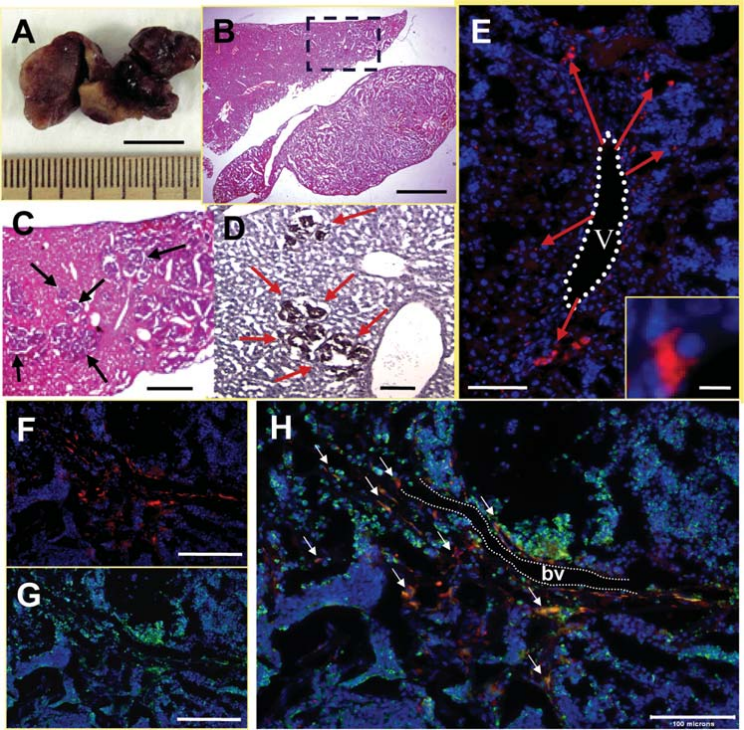
HB1.F3.C1 cells injected intravenously localize to micrometastatic human NB-1643 neuroblastoma tumors in the liver of a mouse. (A) Dissected liver from a representative animal with hepatic metastases; the animal was sacrificed two days following injection of HB1.F3.C1 cells into the tail vein. (B) Low- and (C) high-power magnification of a section of tumor-involved liver, stained with hematoxylin and eosin. Tumor cells appear dark purple (black arrows); normal tissue appears pink. (D) Liver section stained with an anti-human mitochondrial protein antibody and counterstained with hematoxylin. Tumor micrometastases stain dark brown (red arrows); normal liver tissue is purple. (E) Immunofluorescence microscopy of liver section. HB1.F3.C1 cells were CM-DiI-labeled prior to injection and are evident as red cells. The liver section was stained with DAPI; tumor foci are identified by areas of densely-packed DAPI-stained tumor cell nuclei. The red arrows show extravasated HB1.F3.C1 cells proximal to a hepatic vein (v). Inset is high magnification of a DiI-labeled HB1.F3.C1 cell within the tumor. (F–H) Liver section from a tumor-bearing animal that received CM-DiI-labeled HB1.F3.C1 cells was stained with FITC-conjugated human specific mitochondrial antibody. (F) Red CM-DiI-labeled HB1.F3.C1 cells. (G) The same section showing FITC-labeled (green) human tumor cells and HB1.F3.C1 cells. (H) Overlay of F (red CM-DiI HB1.F3.C1 cells) and G (green FITC HB1.F3.C1 and tumor cells). HB1.F3.C1 cells (orange/yellow cells indicated by white arrows) migrated to hepatic micrometastases (green cells) and infiltrated the tumor parenchyma in the proximity of a blood vessel (bv, white dotted lines). Scale bars: 1 cm (A), 2 mm (B), 500 µm (C), 200 µm (F, G), 100 µm (D, E, H), 10 µm (E inset).

**Figure 4:**
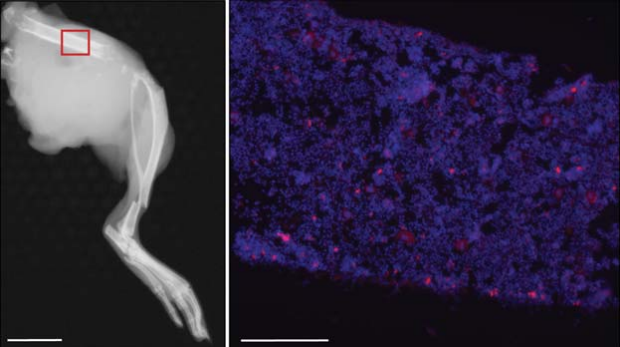
HB1.F3.C1 cells target macroscopic metastatic neuroblastoma in the bone marrow. X-ray image of hind limb of a mouse with advanced stage neuroblastoma (Day 82; left panel, scale bar: 1 cm). Confirmation of the tumor mass as human SK-N-AS neuroblastoma cells was performed by immunohistochemistry using anti-human mitochondria antibody (not shown). The CM-DiI-labeled HB1.F3.C1 cells (red cells, injected into the tail vein 3 days prior to sacrifice) localized to tumor in the marrow (right panel; scale bar: 200 µm).

**Figure 5:**
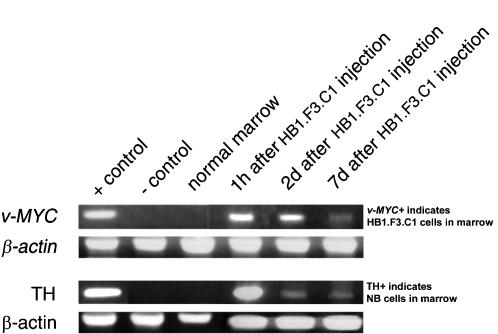
HB1.F3.C1 cells injected intravenously localized to microscopic bone marrow disease. Concordant detection of v-*myc* (HB1.F3.C1 cells) by PCR and TH expression (neuroblastoma cells) by RT-PCR in bone marrow specimens. Bone marrow samples isolated from animals injected with HB1.F3.C1 cells were analyzed for the presence of v-*myc* (HB1.F3.C1 cells) or the expression of TH (NB-1643 cells). HB1.F3.C1 cells were present in the bone marrow only when tumor cells were also present. HB1.F3.C1 cells were not detected in the bone marrow of non-tumor-bearing animals. The positive controls (+) were DNA extracted from HB1.F3.C1 cells for v-*myc*, and RNA extracted from NB-1691 cells for TH. The negative controls (−) contained no DNA or RNA template, respectively.

**Figure 6:**
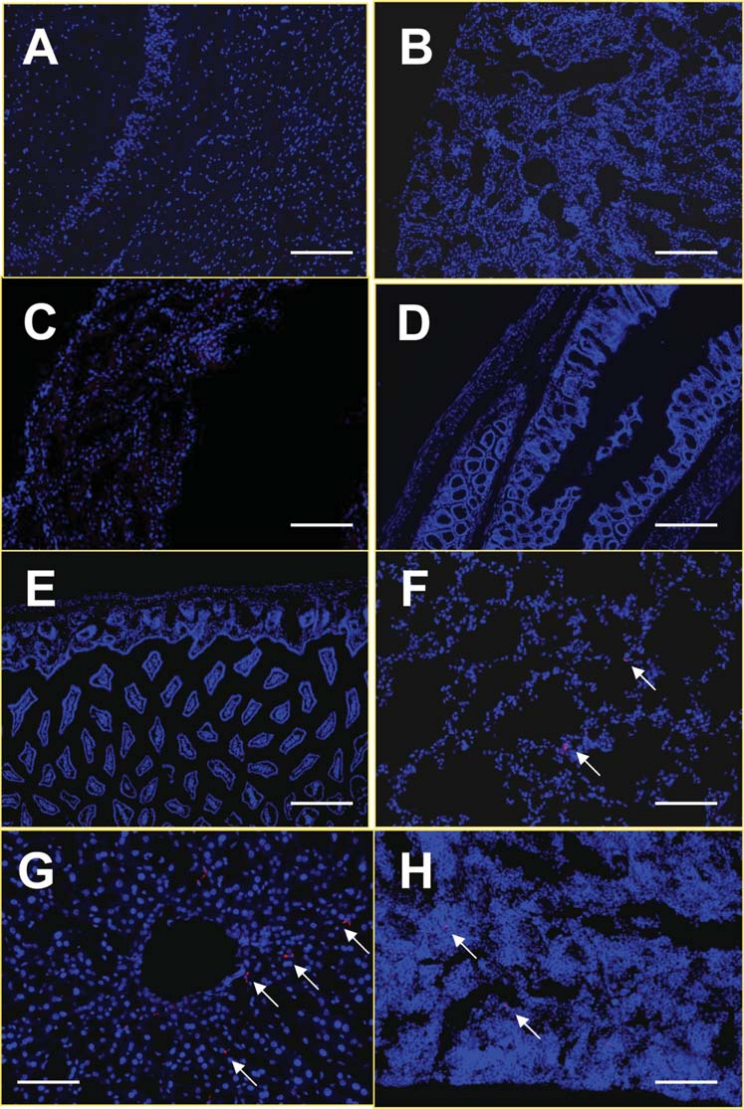
Mice bearing microscopic NB-1643 tumors were injected intravenously with 2 million HB1.F3.C1 cells pre-labeled with CM-DiI Red Cell Tracker. Normal and tumor-bearing organs were harvested, sectioned, and stained with DAPI. HB1.F3.C1 cells were not detected in normal (A) brain, (B) kidney, (C) heart, (D) intestine, or (E) skin tissue. Rare, single, HB1.F3.C1 cells (white arrows) were seen in (F) lung, (G) liver and (H) spleen. Scale bars: 200 µm (A–E), 100 µm (F–H).

**Figure 7:**
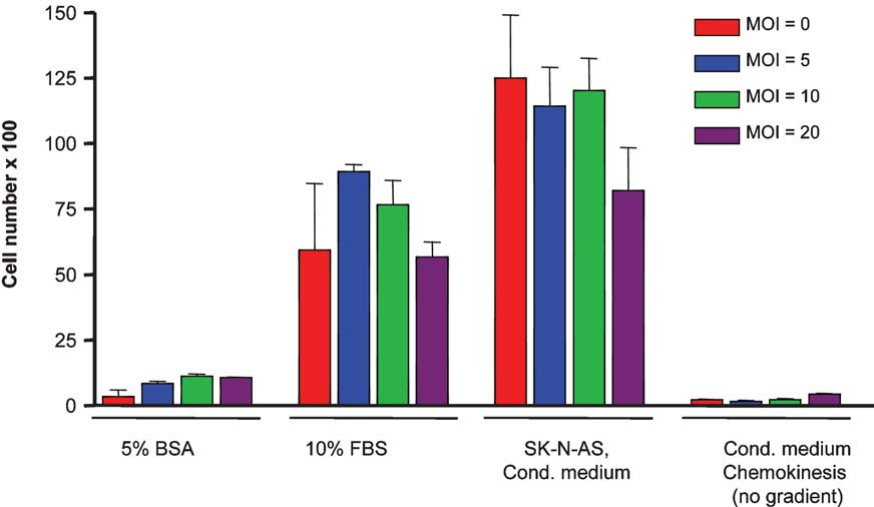
HB1.F3.C1 cells transduced with an adenoviral multiplicity of infection of 5, 10, or 20 retain their tumor-tropism toward conditioned medium from SK-N-AS neuroblastoma cells. Bars represent the average number of migrated cells±SEM of triplicate wells in modified Boyden chamber assays.

**Figure 8:**
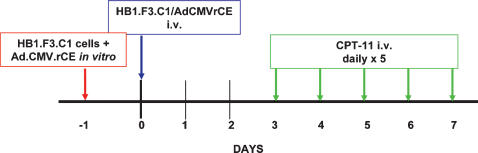
Treatment protocol of HB1.F3.C1/AdCMVrCE/CPT-11 NDEPT. One day prior to being used in treatments, cells were transduced with the AdCMVrCE construct (see text). The treatment schedule was based on the time-course of expression of the secreted form of rCE, following adenoviral transduction of HB1.F3.C1 cells (Danks, unpublished observation) and on a schedule of administration of CPT-11 that has been shown to be relatively effective for neuroblastoma patients [35].

### Statistical Analyses

All data were analyzed with GraphPad Prism software (San Diego, CA). Analyses of cell migration results (mean cell number±S.E.M.) of untransduced cells were compared to that of cells transduced with adenovirus. These data were analyzed using a two-tailed *t*-test. Analysis of Kaplan-Meier plots compared survival of 10 mice per group using a log-rank (Mantel-Haenszel) method.

## Results

### Mouse Model of Metastatic Neuroblastoma

Intravenous injection of 5×10^5^ NB-1691, NB-1643, or SK-N-AS human neuroblastoma cells produces disseminated neuroblastoma in 100% of esterase-deficient SCID mice. The disseminated nature of tumors is evident in Figure 1 in two mice that received tail vein injections of SK-N-AS cells transduced to express luciferase. One month following injection of neuroblastoma cells, the luciferase substrate luciferin was injected intraperitoneally to visualize tumors *in situ*. Consistent with published reports, tumors were present in multiple organs and anatomical locations including abdomen, bone marrow, liver, and lungs [21], [34]. Since 100% of mice injected with neuroblastoma cells develop disseminated disease that ultimately results in death, this model facilitated evaluation of therapeutic experiments which were conducted according to the schema in Figure 2. This Figure illustrates the hypothesis to be tested. Mice injected with human neuroblastoma cells develop multiple tumors. Two weeks following injection of neuroblastoma cells, HB1.F3.C1 cells transduced with adenovirus encoding a prodrug-activating enzyme are administered intravenously. Three days later, after HB1.F3.C1 cells have localized preferentially to tumor sites and the prodrug-activating enzyme (rCE in this study) is expressed at high levels, prodrug (CPT-11) administration is initiated, given on a predetermined optimal schedule. The antitumor efficacy of prodrug alone can then be compared with co-administration of HB1.F3.C1 cells and prodrug, using survival as an endpoint.

### Localization of HB.F3.C1 Cells to Neuroblastoma Tumor in Liver

To first confirm that HB1.F3.C1 cells migrate to and localize at disseminated neuroblastoma tumors, mice with established NB-1643 tumors were injected intravenously with 2×10^6^ CM-DiI labeled HB1.F3.C1 cells. After 3 days, normal and tumor-bearing organs were harvested and sectioned, and evaluated microscopically. As shown in Figure 3, multiple tumors were evident by gross inspection in the liver of a representative mouse (panel A), and also by microscopic evaluation of hematoxylin/eosin stained sections (panels B and C, purple areas) and immunoperoxidase staining for human mitochondrial protein (panel D, brown areas). HB1.F3.C1 cells co-localized with these tumor foci, as demonstrated by the CM-DiI-labeled, red cells seen in panel E. This panel also shows extravasation of the HB1.F3.C1 cells and their migration away from the hepatic vein (v). Panel F shows HB1.F3.C1 NSCs (CM-DiI-labeled, red) and panel G shows same section stained with human mitochondrial antibody-FITC (human NSCs and tumor cells, green). Panel H, an overlay of panels F and G, demonstrates co-localization of HB1.F3.C1 cells (orange/yellow, indicated by white arrows) and tumor cells (green), at sites immediately adjacent to and distant from the blood vessel (bv). Similar results were observed for tumors in multiple other organs including the ovary (not shown) and bone marrow (Figure 4).

### HB1.F3.C1 Cells Localize to Neuroblastoma Metastases in Bone Marrow

Since bone marrow is a frequent site of neuroblastoma metastasis and marrow aspirates or biopsies are used to document the stage of the disease in high-risk patients, we sought to determine whether HB1.F3.C1 cells also infiltrated tumors at this site. The left panel of Figure 4 shows the x-ray of the femur of mouse bearing a macroscopic tumor that originated in the bone marrow. This animal was injected intravenously with CM-DiI-labeled HB1.F3.C1 cells and euthanized three days later. The area of the bone indicated by the red rectangle was then processed for immunofluorescence microscopy. The right panel of Figure 4 clearly shows HB1.F3.C1 cells (red fluorescence) infiltrating the tumor-bearing marrow, documenting that these cells migrated to and co-localized with macroscopic tumors (Figure 4) as well as microscopic tumors (Figure 3). However, while interesting, we considered it more important and relevant to demonstrate migration of HB1.F3.C1 cells to microscopic tumors in bone marrow (Figure 5), as disease at relapse is thought to originate from residual tumor cells present in the marrow or at primary sites. Also, it is essential to the successful clinical application of this approach to show that HB1.F3.C1 cells are not present in normal tissue (Figures 5, 6).

### Molecular Evidence for HB1.F3.C1 Cell and Tumor Cell Co-localization in Bone Marrow

To address the first of these two questions, we evaluated migration of HB1.F3.C1 cells to tumor cells in bone marrow, using a sensitive molecular approach to detect the presence of minimal numbers of tumors cells and/or HB1.F3.C1 cells. To this end, we harvested marrow from mice that had been injected intravenously with NB-1691 cells, followed by HB1.F3.C1 cells two weeks later. As noted above, at this “stage” of disease, tumors are not detectable microscopically. We then used PCR to detect the v-*myc* gene present in HB1.F3.C1 cells and RT-PCR to detect tyrosine hydroxylase (TH), a neuroblastoma cell marker. Data in Figure 5 show that either both v-*myc* and TH or neither of these markers were detected in any given marrow. If the marrow contained tumor cells as indicated by a detectable signal for TH, HB1.F3.C1 cells (a positive signal for v-*myc*) were also present. Conversely, when no neuroblastoma cells were detected, HB1.F3.C1 cells were similarly undetectable. This “co-localization” occurred within 1 hr after injection and persisted for at least 7 days. These data are consistent with results in Figures 3 and 4, and show that HB1.F3.C1 cells rapidly migrate to tumor cells *in vivo*.

### Few HB1.F3.C1 Cells are Present in Normal Tissue

For therapy to be tumor-selective, it was also essential to show that HB1.F3.C1 cells did not migrate to other normal tissues. Accordingly, 2×10^6^ CM-DiI-labeled cells were injected intravenously into mice bearing NB-1643 tumors and after 3 days, all organs were harvested and evaluated for the presence of HB1.F3.C1 cells. Figure 6 demonstrates that few, if any, HB1.F3.C1 cells were present in normal brain, kidney, heart, intestine or skin tissue (panels A–E, respectively). Occasionally, a single cell was observed in the lung, liver or spleen (panels F–H, respectively), but no focus of CM-DiI fluorescence was seen in any normal tissue of any of five mice examined. We concluded that the HB1.F3.C1 cells migrated selectively to neuroblastoma cells following intravenous injection.

### Therapeutic Efficacy for Treatment of Metastatic Neuroblastoma

**Figure 9:**
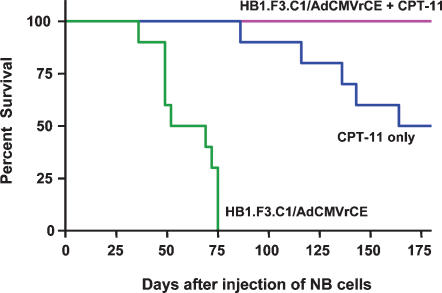
Therapeutic efficacy of rCE/CPT-11 NDEPT. Mice (10/group) received one of the following: 1) 2 million HB1.F3.C1 cells transduced with AdCMVrCE (MOI = 20) encoding a secreted form of rCE; 2) CPT-11 (7.5 mg/kg) alone; 3) transduced HB1.F3.C1 cells and CPT-11 (7.5 mg/kg). Animals that received HB1.F3.C1/AdCMVrCE cells with CPT-11 survived significantly longer than mice receiving only HB1.F3.C1/AdCMVrCE cells (*P*<0.0001) or only CPT-11 (*P*<0.001), suggesting an enhanced tumor targeting and tumor cell-killing effect of CPT-11.

Having established that HB1.F3.C1 cells are tumor-tropic *in vivo*, we evaluated the antitumor efficacy of our neural stem/progenitor cell-directed enzyme prodrug (NDEPT) approach to therapy, using HB1.F3.C1 cells transduced to express a secreted form of a rabbit carboxylesterase (rCE) and the anticancer prodrug CPT-11. We first confirmed, using modified Boyden chamber assays, that adenoviral transduction and expression of rCE did not alter the tropic properties of the HB1.F3.C1 cells (Figure 7). We also determined that the maximal level of expression of rCE occurred 3–4 days following transduction (data not shown). We then transduced HB1.F3.C1 cells with replication-deficient adenovirus encoding rCE, and injected intravenously the transduced cells into mice bearing disseminated SK-N-AS tumors. Mice were treated according to the protocol shown schematically in Figure 8. As shown in the Figure, four days following injection of HB1.F3.C1+AdCMVrCE cells (three days following injection of HB1.F3.C1 cells), we initiated treatment with CPT-11 (7.5 mg/kg) on a daily ×5 schedule, an optimal schedule of administration for patients with neuroblastoma. This treatment was repeated the following week, followed by a 2-week rest period and then another 2-week course of therapy. The Kaplan-Meier graph in Figure 9 shows survival data of untreated mice compared with mice treated with CPT-11 only, or with the combination of HB1.F3.C1 cells expressing rCE and CPT-11. As expected, untreated animals required euthanasia between days 36 and 77 (median 63 days). CPT-11 treatment alone had some antitumor efficacy, with 50% of this group of animals surviving for the 6-month observation period (median survival 169 days, *P*<0.0001 compared to untreated animals). In contrast, 100% of mice treated with rCE/CPT-11 NDEPT, survived without evidence of disease (*P*<0.001 and 0.001, compared to CPT-11 alone or to untreated mice, respectively). These results are consistent with the hypothesis that enhanced drug activation occurred at the disseminated tumor loci within these animals, increasing the anti-tumor efficacy of the CPT-11 and prolonging disease-free survival.

## Discussion

This report is the first to show that undifferentiated cells of neural origin may have utility for targeted treatment of metastatic solid tumors. The described approach relies upon the tumor-tropic property of the HB1.F3.C1 cell line to selectively deliver a prodrug activating enzyme (rCE) to metastatic foci. We propose that when the prodrug (in this instance, CPT-11) is administered systemically, it is preferentially activated at tumor sites leading to high local concentrations of the cytotoxic metabolite and tumor cell death. We evaluated this approach using a preclinical mouse model of the pediatric tumor neuroblastoma, and showed that 100% of animals receiving the described treatment were alive and well 6 months following therapy. Importantly, the approach we describe should be readily translatable into clinical studies, since the dose and schedule of administration of CPT-11 (and the plasma level of active metabolite produced) used in these preclinical experiments are tolerated by patients [35]. We propose that similar methodology may be effective for other types of metastatic tumors, provided the most appropriate stem or progenitor cells are used to deliver therapeutic transgenes that target specific tumor types.

The HB1.F3.C1 cell line used in these studies was derived from the neural cells of the telencephalon of a human fetus of 15 weeks′ gestation, in accordance with the Guidelines for Use of Human Tissue of the University of British Columbia [23]. To maintain these cells in culture, they were immortalized by retrovirus-mediated transduction with the v-*myc* gene. The transduced cell line is non-tumorigenic, as determined by anchorage-independent growth in soft agar or tumor formation in immune-deprived mice [17], [25]. The exact molecular basis of the tumor-tropism of the F3.C1 line or other stem/progenitor cells, is not well understood, but factors such as stromal cell-derived factor-1 (SDF-1; CXCL12), scatter factor (SCF; HGF), vascular endothelial growth factor (VEGF) and macrophage chemotactic protein-1 (MCP-1) expressed by tumor cells likely play a chemotactic role [15], [36]–[42]. Although the mechanism by which HB1.F3.C1 cells target neuroblastoma cells is largely unknown, it is clear that this tropism is not cell line specific since these cells migrated to NB-1643 liver metastases (Figure 3), NB-1691 minimal bone marrow disease (Figure 5), and SK-N-AS macroscopic bone marrow tumors (Figure 4). Studies designed to evaluate the mechanism of tumor cell recognition by stem or progenitor cells are ongoing in our laboratories.

MSCs have also been investigated as potential tumor-selective delivery vehicles, to deliver interferon-β to animal models of MDA-231 breast cancer nodules in the lung [14]. Two concerns that must be addressed before the type of approach described in that study can be incorporated into clinical trials are, first, that MSCs continue to replicate *in vivo* and also incorporate into tumor stroma, constituting a significant fraction of the stromal tissue and possibly supporting tumor growth [43], [44]. In contrast, HB1.F3.C1 cells do not replicate *in vivo* and undergo apoptosis in normal tissue [4], [45]. HB1.F3.C1 cells also differ from bone marrow-derived MSCs in that MSCs engraft in the marrow of recipients [46], whereas HB1.F3.C1 cells were detectable in marrow only if tumor cells were also present (Figure 3A). Additionally, HB1.F3.C1 cells migrate to subcutaneous xenografts of numerous solid tumors including prostate, breast, melanoma, glioma, and neuroblastoma (Aboody et al., unpublished observations). These results suggest that this type of undifferentiated cell of neural origin may be preferable to MSCs when relatively short-term survival of stem/progenitor cells is desirable such as for cancer therapy, and that cells or cell lines similar to HB1.F3.C1 cells may have significant utility for the treatment of metastatic tumors of different histotypes.

The second concern regarding the MSC-mediated therapy with interferon-β is that expression of this transgene increased the median survival of mice from 36 to 60 days. While this was a significant increase, no cures were attained and the duration of survival, even in treated animals, was limited. In contrast, data presented in the current study show that HB1.F3.C1 cell-mediated delivery of an efficient prodrug-activating enzyme produced 6-month disease-free survival in 100% of mice, compared to 0% survival of less than 2 months for untreated mice. This observation emphasizes the necessity for careful selection of the transgene to be delivered to treat a specific tumor type. Our approach using HB1.F3.C1 cells expressing rCE to activate CPT-11 at the tumor site, effectively increasing the therapeutic index of the drug, was based in part on the encouraging but non-curative activity of CPT-11 in early clinical trials of children with neuroblastoma and in part on the inefficient activation of CPT-11 by human enzymes.

Since establishment of the HB1.F3.C1 cell line, it has become clear that stem/progenitor cells can be harvested from many fetal and adult tissues. While published data suggest that the immunogenic potential of cells from allogeneic sources may be minimal [47], further studies are necessary before clinical trials can be initiated. If immunogenicity is determined to be problematic, the use of appropriate immune-suppressive treatment for the time during which viable cells are present *in vivo* would circumvent immune-related limitations of the described approach. Alternatively, it may be possible to identify autologous sources of stem/progenitor cells, such as excess adipose tissue, that could be used for clinical applications.

The prodrug used in this study, CPT-11, is relatively effective for the treatment of many solid tumors including neuroblastoma [35]. Our results demonstrate that the anti-tumor efficacy of CPT-11 was significantly increased when this prodrug was used as a component of NDEPT. Since CPT-11 is effective in the treatment of colon cancer, a logical follow-up study would be to evaluate the anti-tumor efficacy of rCE/CPT-11 NDEPT in a model of residual colon cancer, or of hepatic metastases of this disease. As the level of SN-38 (active metabolite of CPT-11) that is tolerated by patients is known, doses of CPT-11 in preclinical experiments can be restricted to those that produce clinically tolerated levels of SN-38. Such studies may yield results that are readily translated to clinical applications.

Lastly, we propose that NDEPT will have the greatest therapeutic efficacy as an adjuvant treatment to eradicate minimum residual disease, rather than as an initial treatment for reducing bulk tumor volume. This hypothesis reflects both the current paucity of information regarding what ratios of stem or progenitor cells/tumor size are required to attain complete tumor regressions and what ratios are achievable *in vivo*. Furthermore, clinical experience shows that at diagnosis, surgery, chemotherapy, radiation, and bone marrow transplantation are relatively effective even in high-risk neuroblastoma patients. Therefore, we propose that the most logical application of this methodology is during the window in which patients are apparently free from residual disease, but who, based on prognostic indicators, will relapse with metastatic tumors. NDEPT may be a viable option for eradicating undetectable, residual disease. If successful, this treatment might significantly improve the long-term survival of patients diagnosed with high-risk neuroblastoma.
